# Alcohol Availability and Targeted Advertising in Racial/Ethnic Minority Communities

**Published:** 1998

**Authors:** Maria Luisa Alaniz

**Affiliations:** Maria Luisa Alaniz, Ph.D., is a study director at the Prevention Research Center, Berkeley, CA, and an associate professor of social science at San Jose State University, San Jose, California

**Keywords:** AOD availability, advertising, minority group, racial group, violence, societal AODR (alcohol and other drug related) problems, African American, Hispanic, Asian American, California, Louisiana, alcoholic beverage sales outlet, location and density of AOD outlet, poverty, immigrant, prevention campaign, community-based prevention, literature review

## Abstract

Alcohol availability and advertising are disproportionately concentrated in racial/ethnic minority communities. Although research on alcohol availability and alcohol advertising in racial/ethnic minority communities is limited, evidence does show a relationship between minority concentration, alcohol outlet density, and alcohol problems. This article reviews research showing that certain neighborhood characteristics, such as alcohol outlet density, can be stronger predictors of homicide and violence than are race or ethnicity.

Alcohol availability and advertising are disproportionately concentrated in ethnic minority communities. (Alaniz in press; Hackbarth et al. 1995; Altman et al. 1991). Research has shown a direct relationship between alcohol availability (measured by the number of bars, restaurants, and stores selling alcohol in a specific geographical area, such as a city block) and alcohol-related problems, such as violence (Alaniz et al. 1998; Parker and Rebhun 1995; Scribner et al. 1995). Alcohol outlet density also is an important determinant of the amount of alcohol advertising in a community. Community advocates and local policymakers have formed coalitions across the country to limit the quantity, operation, and types of alcohol outlets in their communities. Such coalitions have succeeded in various cities, such as Baltimore, Chicago, south central Los Angeles, and Oakland, California. This article reviews recent research on alcohol availability and advertising in ethnic minority communities and associated alcohol-related problems. In addition, the article discusses one community’s efforts to reduce these problems.

Overall, research on alcohol availability and alcohol advertising in racial/ethnic minority communities is limited. This review focuses on two minority groups, African-Americans and Latinos, in cities in California (Alaniz et al. 1998; Scribner et al. 1995) and Louisiana (Scribner et al. in press). (Alcohol availability and advertising among other groups, such as Asian-Americans, have not been studied at the community level.) In the studies reviewed, alcohol availability is measured either by the number of outlets in a given geographical area (e.g., a group of city blocks) or by the density of outlets in an area (i.e., the number of outlets for every 1,000 residents in an area). Two types of outlets exist: (1) on-site, where alcohol is consumed on the premises, and (2) off-site, where alcohol is purchased for consumption off the premises.

## Alcohol Availability and Alcohol-Related Problems

The degree of alcohol availability and alcohol use in a community affects its residents’ social, physical, and economic well-being (Moore and Gerstein 1981). Watts and Rabow (1983) analyzed the effects of alcohol availability on alcohol-related problems in 213 California cities. They found higher rates of alcohol-related problems in areas with higher alcohol outlet densities compared with areas with lower alcohol outlet densities. Specifically, Watts and Rabow (1983) reported that a 1-percent increase in the density of “beer bars” (e.g., on-site establishments that only serve beer) resulted in a greater than 1-percent increase in the arrest rates for public drunkenness and misdemeanor drunken driving. In addition, they found higher alcohol outlet densities in communities with higher proportions of African-American, other nonwhite, or foreign-born residents. The relationship between minority concentration, alcohol outlet density, and alcohol problems is further defined by three recent studies (Alaniz et al. 1996; Alaniz et al. 1998; Scribner et al. in press), discussed next, which show that certain neighborhood characteristics, such as alcohol outlet density, can be stronger predictors of homicide and violence than are race or ethnicity.

The first study, conducted in three northern California cities, examined the relationship between alcohol outlet density and Latino youth violence (Alaniz et al. 1996). The researchers created maps of the cities to reflect (1) alcohol outlet density (i.e., the number of outlets for every 1,000 residents in an area), (2) ethnic or racial concentration, and (3) financial status. The maps showed that geographic areas with high concentrations of poor racial or ethnic minorities (i.e., where ethnic or racial minorities in poverty make up at least one-half of the population) tend to have high alcohol outlet densities (i.e., higher than the statewide average). The researchers examined individual “block groups” (i.e., four city blocks or the equivalent) to approximate neighborhoods (see figure, p. 288). For example, in one of the three cities, 53 percent of the population was white, yet only two block groups had a concentration of alcohol outlets, poverty, and whites (i.e., in only two block groups whites in poverty made up more than one-half of the population and the alcohol outlet density was higher than the statewide average). By comparison, 29 block groups were found to have a high concentration of Latinos, poverty, and alcohol outlets, although Latinos made up only 29 percent of the population. A high concentration of Vietnamese-Americans, alcohol outlets, and poverty was found in nine block groups, and Vietnamese-Americans made up only 13 percent of the city’s population. Thirteen block groups had a high concentration of African-Americans, alcohol outlets, and poverty, even though African-Americans made up only 4 percent of the population. This analysis shows an overconcentration of alcohol outlets in poor racial or ethnic communities. Even though the majority of the population was white, only two block groups of predominantly white residents had a high concentration of alcohol outlets.

### Alcohol Availability and Violence

In a second study, in which data from each of the three cities were further analyzed, Alaniz and colleagues (1998) found that the violent crime arrest rate for Latino youth ages 15 to 24 was significantly and positively related to the number of alcohol outlets (i.e., crime increased with the number of outlets), independent of the neighborhood’s social and economic characteristics (Alaniz et al. 1998). In neighborhoods with no alcohol outlets, the arrest rate for Latino youth was 1.19 arrests for every 1,000 compared with 2.67 arrests per 1,000 in neighborhoods with at least one alcohol outlet. The youth violence rate in the block groups with the highest concentration of alcohol outlets was 18.67 arrests per 1,000. The percentage of divorced adults in the community also was significantly and positively related to youth violence. The percentage of professionals in the community had both a negative and significant effect: as the number of professionals (i.e., positive role models) increased, the incidence of youth violence decreased. The percentage of other immigrants, Latinos, or African-Americans in the community had no effect on youth violence.

The authors propose two theories to explain the relationship between alcohol availability and youth violence. One theory suggests that youth living in areas with high concentrations of alcohol outlets are more likely to drink and therefore more likely to commit crimes. The other theory suggests that young people tend to congregate in areas where alcohol outlets are concentrated, because these areas are characterized by relaxed social restrictions and a lack of monitoring. The authors conclude that in order to understand the relationships between alcohol availability, race or ethnicity, immigration status, and violence, all of the neighborhood’s characteristics must be examined.

In a third study, recently conducted in New Orleans, a city with a high concentration of African-Americans, Scribner and colleagues (in press) found that the concentration of alcohol outlets in a neighborhood, especially off-site outlets, was a major predictor of homicide rates. A neighborhood with two off-site outlets was found to have a homicide rate 24-percent higher than a comparable neighborhood with one off-site outlet. The results are independent of other neighborhood characteristics, such as the percentage of African-American, unemployed, young male residents and the percentage of single-parent families. The findings demonstrate that the concentration of alcohol outlets is a stronger predictor of violence than is race or ethnicity in racially/ethnically segregated areas.

In the three studies, the researchers used an analysis of alcohol availability rather than measures of alcohol use by the population to demonstrate that environmental factors affect levels of violence in a neighborhood. The theories and data described previously (Alaniz et al. 1998) suggest that individual and environmental factors interact to perpetuate alcohol-related problems.

## Targeted Advertising

Alcohol outlet density is an important determinant of the amount of alcohol advertising in a community. Merchants use storefronts and the interiors of alcohol outlets to advertise alcohol products. Therefore, areas with a high density of outlets have a greater number of advertisements. One study found that a student walking from home to school in a predominantly Latino community in northern California may be exposed to between 10 and 60 storefront alcohol advertisements (Alaniz and Wilkes 1995). The same study found that there are five times more alcohol advertisements in Latino neighborhoods than in predominantly white neighborhoods. Exposure to alcohol advertising on television has been linked to children’s attitudes toward alcohol. Grube and Wallack (1994) found that children’s exposure to beer commercials and their ability to recall the brands advertised were associated both with saying that they would drink as adults and with holding positive beliefs about the social uses of beer. Research has not established whether exposure to alcohol advertising during childhood is associated with later drinking. Nevertheless, the implications of these findings may be especially problematic for ethnic minority children because they are exposed to advertisements not only on television but also in their immediate neighborhood environment.

### Billboard Advertising

Studies of alcohol advertising in ethnic minority communities primarily have focused on billboards. A San Francisco-based study found that African-American and Latino neighborhoods had proportionally more billboards advertising alcohol and tobacco than white or Asian neighborhoods (Altman et al. 1991). Thirty-one percent of the billboards in Latino neighborhoods advertised alcohol, compared with 23 percent in African-American neighborhoods, 13 percent in white neighborhoods, and 12 percent in Asian neighborhoods. Most of the alcohol billboards in the Latino community advertised beer and wine, whereas the majority of billboards in the African-American community advertised malt liquor and distilled spirits. A survey of billboards in St. Louis found twice as many billboards in African-American neighborhoods compared with white neighborhoods. Almost 60 percent of the billboards in the African-American neighborhoods advertised either tobacco or alcohol (American Public Health Association Governing Council 1993). In a study of 50 Chicago neighborhoods, Hackbarth and colleagues (1995) found an average of 7 alcohol billboards in white neighborhoods and an average of 38 in minority neighborhoods.

## Community Response

Ethnic and racial groups have historically organized grass-roots antialcohol movements to prevent alcohol-related problems in their communities (Duran and Duran 1995; Herd 1985). Many such movements in communities across the United States have targeted the high concentration of alcohol outlets and billboard advertising in racial and ethnic neighborhoods.

For example, a 1993 study found that West Oakland—an area in Oakland, California, in which racial and ethnic minorities and the poor are concentrated—had 1 liquor outlet for every 298 residents. By comparison, Piedmont, the more affluent, predominantly white area of Oakland, had 1 alcohol outlet for every 3,000 residents (Mack 1997). In 1993 concerned citizens formed the Coalition on Alcohol Outlet Issues (CAOI) in an effort to reduce the problems associated with alcohol outlet concentration in Oakland’s minority neighborhoods. The primary purpose of the CAOI was to reduce the density of alcohol outlets. The group’s efforts led to the passage of an ordinance creating the Education, Monitoring and Enforcement Program. This program established operating standards for all city alcohol outlets (Mack 1997). It mandated that alcohol outlets “avoid creating a public nuisance, endangering public health or safety, or violating criminal laws” (Mack 1997). If a merchant was found liable, specific conditions were placed on the operation of the outlet. If the conditions were violated, the alcohol outlet was no longer considered “approved” by the city. Based on the success of the pilot program, this ordinance has now been implemented citywide.

## Summary

Research reviewed in this article suggests that structural neighborhood characteristics (i.e., alcohol outlet density) can be stronger predictors of alcohol-related problems than the population’s racial or ethnic makeup. Furthermore, there is a disproportionate concentration of alcohol outlets and advertisements in low-income minority communities.

**Figure:**
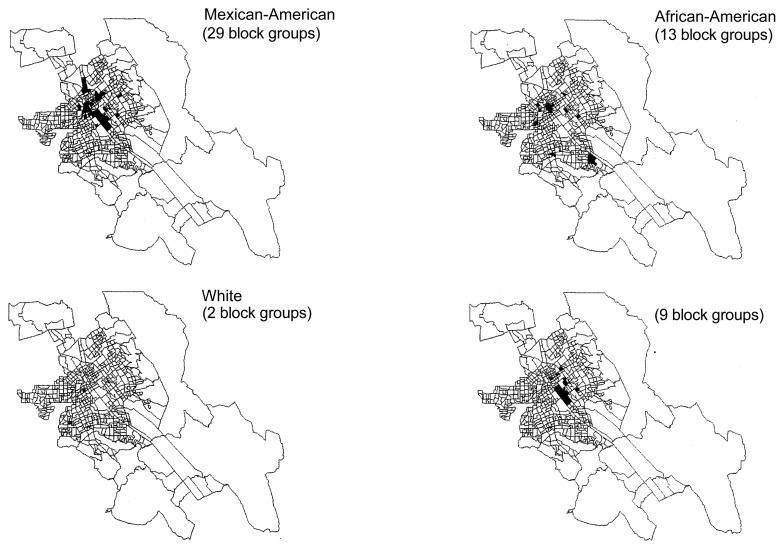
Overlap of high poverty areas with high outlet densities and high densities of four ethnic groups.

Low-income minority communities are aware of the problems associated with alcohol availability and alcohol advertising. Some communities are successfully combating these problems. The success of one coalition in Oakland, California, demonstrates the ability of concerned citizens to work together to improve their community.
